# Engineering a T7 bacteriophage to attenuate LPS-driven inflammatory responses during bacteriolysis

**DOI:** 10.1128/aem.02303-25

**Published:** 2026-05-04

**Authors:** Tong Yu, Junjiao Pang, Mengge Chen, Qi Sun, Jiaqi Pu, Deshu Wang, Qingling Liu, Fengtang Yang, Hongkuan Deng

**Affiliations:** 1 School of Life Sciences and Medicine, Shandong University of Technology 91620, Zibo, Shandong, China; 2 Department of Clinical Laboratory, Zibo Maternal and Child Health Hospital 671977, Zibo, Shandong, China; Universidad de los Andes, Bogotá, Colombia

**Keywords:** engineered bacteriophage, bacteriophage therapy, alkaline phosphatase, inflammatory factor

## Abstract

Bacterial lysis during treatment of Gram-negative infections can release lipopolysaccharide (LPS) and aggravate inflammation. Here, we engineered two complementary T7 bacteriophages: T7-*nluc*, a NanoLuc reporter bacteriophage for real-time monitoring of viable bacteria, and T7-*phoa*, a therapeutic bacteriophage that releases alkaline phosphatase (PhoA) during lysis to reduce LPS bioactivity. Both engineered bacteriophages retained lytic activity similar to that of wild-type T7. *In vitro*, T7-*nluc* produced a low-background bioluminescent signal that reflected bacterial burden, whereas T7-*phoa* released catalytically active PhoA into the extracellular environment. In *Galleria mellonella* and *Danio rerio* infection models, T7-*nluc* enabled dynamic monitoring of infection progression, while T7-*phoa* improved survival, reduced inflammatory responses, and accelerated inflammatory resolution without compromising bacterial clearance. These findings support a modular bacteriophage engineering strategy that combines bacterial killing, real-time infection monitoring, and local attenuation of LPS-driven inflammation, offering a potential approach for improving bacteriophage-based treatment of Gram-negative infections.

**IMPORTANCE:**

Bacteriophage therapy is being reconsidered for treating drug-resistant Gram-negative infections, but there is concern that rapid bacterial lysis may release LPS and worsen inflammation. We used bacteriophage T7 as a platform to test whether bacteriophages can be engineered to both fight bacteria and soften these harmful host responses. First, we created a NanoLuc reporter bacteriophage that produces light only when it grows in live bacteria, confirming that engineered bacteriophages can deliver active proteins directly in infected animals. We then built a therapeutic T7-*phoa* bacteriophage designed to release enzymatically active alkaline phosphatase upon on-target lysis, thereby providing lysis-coupled local phosphatase activity at the infection site. In both *G. mellonella* and *Danio rerio* models, infection-site fluids collected after treatment showed elevated phosphatase activity in the T7-*phoa* group, and the treatment was associated with lower inflammatory peaks, improved survival, and preserved bacterial clearance. Together, these results support a modular route for bacteriophage-based strategies that couple bacterial killing with real-time reporting and local control of LPS associated inflammation.

## INTRODUCTION

Antimicrobial resistance has made Gram-negative infections an increasing cause of morbidity and mortality (1). Lipopolysaccharide (LPS) released during bacteriolysis propagates inflammatory signaling through the TLR4-NF-κB pathway (2). The ensuing cytokine surge compromises epithelial tight junctions, increases paracellular permeability, and aggravates tissue injury and systemic sequelae (3).

Alkaline phosphatase (PhoA) removes phosphate groups from the lipid A of LPS, blunting TLR4 activation and curbing pro-inflammatory cytokine production (4). It also dephosphorylates extracellular ATP/ADP, tempering P2 receptor-dependent feed-forward amplification (5).

These enzymatic activities confer combined endotoxin-neutralizing and anti-inflammatory effects. In cell-based systems, exogenous PhoA under LPS challenge markedly reduces IL-6, IL-1β, and TNF-α transcripts while upregulating tight-junction determinants such as ZO-1 and CLDN-3, thereby stabilizing mucosal barrier function (6). Collectively, these data support the premise that sustained, *in situ* delivery of catalytically competent PhoA at infection sites could attenuate LPS-driven inflammation, limit barrier failure, and mitigate secondary injury.

Bacteriophage therapy has reemerged as a compelling option against antimicrobial resistance because of its host specificity and self-amplifying replication (7, 8). Bacteriophages initiate infection through defined interactions with bacterial surface receptors (9, 10), which confers selectivity at the genus, species, and strain levels. Their capacity to replicate at the site of infection, together with low direct toxicity to nontarget cells and compatibility with antibiotics (11, 12), makes bacteriophage therapy a promising strategy for managing infections that are resistant to multiple drugs and for correcting microbial dysbiosis. Importantly, mitigating excessive inflammation is directly beneficial for animal hosts. In preclinical and veterinary contexts, attenuating the inflammatory surge can lower morbidity and stabilize performance traits, thereby improving overall health status (13–15). This creates a strong rationale to equip bacteriophages with anti-inflammatory payloads alongside their lytic function. Beyond therapy, the strict requirement for viable hosts has been exploited diagnostically: reporter bacteriophages engineered to carry heterologous genes generate signals only after successful infection and replication, enabling rapid readouts when the appropriate substrate is present (16–18). Among available reporters, NanoLuc luciferase (NLuc) is well suited to low biomass samples and complex matrices because of its small size, minimal background, independence from ATP, and high photon output (19, 20).

Advances in synthetic biology now support iterative bacteriophage engineering, including yeast-based capture and reboot of complete genomes, modular insertion of expression cassettes (21), rational editing of host range determinants (22), and loading of effector payloads such as cell wall hydrolases, toxins, and immunomodulators (17, 23). Together, these capabilities provide bacteriophages with dual functionality by combining potent lytic activity with *in situ* delivery of therapeutic effectors.

Guided by the principle that moderating host inflammation benefits animal health outcomes, we combined real-time infection readout with *in situ* detoxification. In this study, we designed and validated an engineered bacteriophage strategy that integrates *in situ* visualization with *in situ* detoxification. A bioluminescent reporter gene (*nluc*) and an inflammation-modulating effector gene (*phoa*) were inserted separately into the T7 chassis and placed under bacteriophage promoters to ensure expression during the infection and replication cycle. The encoded proteins are released upon host lysis. NLuc provides an *in vivo* readout of ongoing infection in real time, whereas PhoA is designed to provide lysis coupled local phosphatase activity at the infection site, which is consistent with attenuated LPS-driven inflammatory signaling, while preserving pathogen clearance. This design improves overall therapeutic performance and aims to reduce reliance on rescue antimicrobials, thereby increasing treatment efficiency.

## MATERIALS AND METHODS

### Bacterial strains, vector, and primers

Laboratory strains of *Escherichia coli*, including *E. coli* 10G (Lucigen, USA) and *E. coli* BL21 (NEB, USA), were used as hosts for bacteriophage propagation and genome engineering. *E. coli 65* is a clinical isolate obtained from Zibo Maternal and Child Health Hospital (Zibo, Shandong, China) and was recovered from a urine specimen. *E. coli 65* is resistant to representative third-generation cephalosporins and fluoroquinolones. The detailed resistance profile is provided in Data S2. All *E. coli* strains were grown at 37°C in Luria-Bertani (LB) broth. *Saccharomyces cerevisiae* BY4741 (Coolaber, China) was cultured at 30°C in YPD medium (1% yeast extract [BD], 2% peptone [BD], 2% dextrose [VWR]). The pRS415 centromeric yeast vector carrying the LEU2 marker (ATCC 87520) was obtained from HonorGene. Primer sequences are provided in Data S1.

### Preparation of bacteriophage genomes

High titer bacteriophage stocks were propagated in *E. coli* BL21. Briefly, 100 µL of bacteriophage suspension (10⁹−10¹⁰ PFU/mL) was added to 1 mL of exponentially growing bacterial culture (OD₆₀₀ = 0.5), resulting in a multiplicity of infection (MOI) of 0.01–0.1. The infection mixture was incubated at 37°C with gentle shaking for 3 h until complete bacterial lysis was observed. The lysate was then clarified by filtration through a 0.22 µm membrane filter. The filtrate was treated with DNase I (10 µg/mL) and RNase A (1 U/10 mL) at 37°C for 30 min to remove residual host nucleic acids. Bacteriophages were concentrated by polyethylene glycol (PEG) precipitation. PEG 8000 and NaCl were added to final concentrations of 10% (wt/vol) and 1 M, respectively, and the mixture was incubated overnight at 4°C. The bacteriophage pellets were collected by centrifugation at 12,000 × *g* for 30 min at 4°C and resuspended in 1 mL of PBS. Subsequently, 10 μL of 10% SDS was added and incubated the samples at 70°C for 20 min. An equal volume of phenol:chloroform:isoamyl alcohol (25:24:1, Sigma, USA) was added to the sample, followed by centrifugation at 12,000 × *g* for 10 min at 4°C. The upper aqueous phase was transferred to a new tube, mixed with 1/10 volume of sodium acetate (3 M, pH 5.2) and 2 volumes of absolute ethanol, and incubated at −20°C for 2 h. The mixture was centrifuged at 12,000 × *g* for 10 min at 4°C to pellet DNA. The pellet was washed with 70% ethanol and centrifuged at 12,000 × *g* for 2 min at 4°C. The supernatant was carefully removed, and the tube was air-dried at room temperature. The DNA pellet was then resuspended in an appropriate volume of water. DNA concentration was quantified using a Qubit fluorometer.

### Preparation of PCR products for assembling bacteriophage genomes

All PCR products were prepared with specific primer sets (Data S1) and DNA Polymerase. Eight 3.11–10 kb PCR products including the YAC amplicon were used per reaction. Homology arms between the YAC and the bacteriophage genome were added to the first and last bacteriophage genome fragments. When capturing bacteriophage genomes from genomic DNA, the YAC amplicon was gel extracted to reduce background.

### Preparation of yeast competent cells

The preparation of yeast competent cells was performed as described by Ando et al. (21), with minor modifications. Briefly, *S. cerevisiae* BY4741 was grown in 10 mL YPD at 30°C for approximately 24 h. Five milliliters of this culture was transferred into 50 mL of YPD and incubated at 30°C for 4 h. Cells were harvested and washed with 1 mL of 100 mM lithium acetate (LiAc) (Orileaf, China) and suspended in 400 μL of 100 mM LiAc. One hundred microliters of cell suspension was used for each transformation.

### Yeast transformation

Yeast transformation was performed according to a previously described protocol with minor modifications (21). Bacteriophage DNA fragments and the linearized vector pRS415 were pooled in a tube (1 µg of each fragment plus 200 ng of linearized pRS415 in 35 µL of water). The DNA mix was combined with a transformation cocktail consisting of 100 µL competent yeast cells, 240 µL of 50% (wt/vol) PEG 3350, 36 µL of 1 M LiAc, and 50 µL of salmon sperm DNA (2 mg/mL; Sigma, USA). After thorough mixing, the suspension was heat-shocked at 42°C for 45 min, pelleted by centrifugation (13,000 × *g* for 30 s), and resuspended in 200 µL water. Cells were spread onto SD-Leu selective agar plates (0.67% Yeast Nitrogen Base [YNB], 0.069% CSM-Leu, 2% dextrose) and incubated at 30°C for up to 4 days to recover transformants.

### Yeast DNA extraction of captured bacteriophage genomes

YAC-bacteriophage DNA was extracted from yeast cells as previously described (21).

### Rebooting of bacteriophages

Bacteriophages were initially propagated using *E. coli* 10G as the host. Three microliters of purified bacteriophage DNA was electroporated into 50 µL of electrocompetent cells in a 2 mm gap cuvette at 2,500 V, 25 µF, and 200 Ω (Bio-Rad, USA). Immediately after pulsing, the cells were mixed with 1 mL of Recovery Medium (Lucigen, USA) and incubated at 37°C with shaking at 150 rpm for 1 h. Three hundred microliters of the culture was mixed with 3 mL of LB soft agar prewarmed to 42°C, overlaid onto LB agar plates, and incubated at 37°C for 3 h to allow plaque formation.

### Bacteriophage activity assays

#### Spot-on-the-lawn assay

Two hundred microliters of stationary-phase bacterial culture was mixed with 3 mL of molten LB soft agar and overlaid onto an LB agar plate. Once solidified, serial dilutions (2 µL) of bacteriophages with titer between 10 and 10^6^ PFU/mL were spotted onto the bacterial lawn, incubated for 12 h at 37°C, and inspected for plaque formation.

#### Turbidity reduction assays

Log-phase cultures were diluted in LB to an OD₆₀₀ of 0.1–0.2, dispensed into clear, flat-bottom 96-well plates (Corning, USA), and challenged with bacteriophage to a final titer of 1 × 10⁴ PFU/mL. OD₆₀₀ was recorded every 15 min at 37°C on a microplate spectrophotometer (SpectraMax iD5 Molecular Devices). Parallel wells containing bacteria without bacteriophage served as growth controls, and medium-only wells served as background/sterility controls. Each condition was run in technical triplicate, with results are reported as mean ± SD. Data processing and statistical analyses were performed in GraphPad Prism (v8).

### Bioluminescence assay and PhoA activity assay

In the logarithmic growth phase, *E. coli 65* was infected with T7-*nluc*, T7-*phoa*, or wild-type T7 (MOI = 0.1) and incubated at 37°C for 3 h. The culture was then centrifuged (4°C, 4,000 rpm, 10 min) to obtain cell-free supernatants and bacterial pellets. The bacterial pellets were resuspended in LB. To compare the bioluminescence in the supernatants and pellets of T7-*nluc* and wild-type T7, 200 µL of supernatant and 200 µL of resuspended bacterial pellets were each mixed with 10 µL of pre-diluted NLuc substrate (Furimazine) in a white opaque 96 well plate. After incubating the plate in the dark at room temperature for 5 min, bioluminescence was immediately read in top-read mode with a 0.5 to 1.0 s integration and background subtraction. Meanwhile, PhoA activity in the supernatants and resuspended bacterial pellets from T7-*phoa* and T7 infections was quantified using a commercial alkaline phosphatase assay kit (Beyotime, China) according to the manufacturer’s instructions.

### *In vivo* bioluminescence imaging of T7-*nluc* in *G. mellonella* and zebrafish (*Danio rerio*)

Larval *G. mellonella* and adult zebrafish were used for *in vivo* bioluminescence imaging on a Tanon Prime 2000XDM. Larvae were assigned to four groups (bacteria + T7 *nluc*, bacteria + T7, bacteria + PBS, PBS + PBS). A total of 10 µL of 1 × 10^7^ CFU/mL *E. coli* or PBS was injected into the last left proleg, followed 1 h later by 10 µL of T7-*nluc*, T7, or PBS (1 × 10^9^ PFU/mL) into the last right proleg. At 6, 12, 18, and 24 h, 2 µL furimazine was injected into the last right proleg, and after 5–10 min photon flux was quantified by ROI analysis under fixed acquisition settings. Adult AB strain zebrafish (approximately 3–6 months old, 0.3–0.6 g) were maintained under standard husbandry conditions in a recirculating aquatic system (28°C, 12 h light and 12 h dark photoperiod). Water quality was monitored and maintained within standard ranges (pH 7.0 to 7.5 and conductivity 500 µS/cm), with routine filtration and partial water renewal. Fish were fed *ad libitum* three times daily and acclimated for at least 7 days prior to experiments. Fish received an intraperitoneal injection of 10 µL of *E. coli 65* (1 × 10^7^ CFU/mL) or PBS, followed 6 h later by an intraperitoneal injection of 10 µL of T7-*nluc* (1 × 10^9^ PFU/mL). Control fish received T7 or PBS. At 6, 12, 18, and 24 h after bacterial injection, 10 µL NLuc substrate was administered intraperitoneally, and bioluminescence was recorded 5 min later with identical instrument settings. All experiments were performed in at least three independent biological replicates.

### Effectiveness of bacteriophage therapy on *G. mellonella* and zebrafish infection model

For the *G. mellonella* infection experiments, an overnight culture of *E. coli 65* was harvested, washed twice with PBS, and adjusted to 1 × 10^7^ CFU/mL. Larvae (non-pigmented, 300–400 mg) were stored at 17°C and used within 7 days. Before inoculation, each larva was surface sterilized with 70% ethanol. Using a 10 µL Hamilton syringe with a blunt-tip needle (Merck, Germany), each larva was injected with 10 µL into the last left proleg according to group assignment. One hour later, a second 10 µL injection was administered into the last right proleg. Animals were randomized into four groups (*n* = 15 per group): (i) PBS control, 10 µL PBS (left proleg), followed by 10 µL PBS (right proleg). (ii) Infection control, 10 µL *E. coli 65* suspension (1 × 10^5^ CFU), then 10 µL PBS. The 1 × 10^5^ CFU challenge caused substantial mortality in untreated controls by 24 h, yet allowed clear bacteriophage rescue over 48 h. (iii) Bacteriophage control, 10 µL *E. coli 65* (1 × 10^5^ CFU), then 10 µL wild-type T7 in PBS delivering 1 × 10^7^ PFU (MOI = 100 relative to the bacterial dose). (iv) T7-*phoa* therapeutic, 10 µL *E. coli 65* (1 × 10^5^ CFU), followed by 10 µL T7-*phoa* in PBS delivering 1 × 10^7^ PFU (MOI = 100). Following injection, larvae were incubated at 37°C in the dark for 48 h, and survival was recorded every 6 to 12 h intervals. Death was defined as complete melanization with no response to touch. Kaplan-Meier survival curves were generated in GraphPad Prism (v8). For PhoA activity measurement, an independent subset of larvae (*n* = 6 per group) was sampled at 1 h post-bacteriophage treatment. Hemolymph was collected into pre-chilled tubes, clarified by centrifugation at 4°C to remove cells and debris, and the supernatant was subjected to an alkaline phosphatase activity assay. For the zebrafish infection experiments, zebrafish were randomized into the same four groups as in the *G. mellonella* experiment (*n* = 15 per group): PBS control, infection control, wild-type T7, and T7-*phoa*. Fish were challenged with 1 × 10^5^ CFU of *E. coli 65*, except for the PBS control group, which received PBS as the baseline control. The same challenge dose was used as in the *G. mellonella* experiments. After 6 h, fish were treated according to group assignment: PBS control, PBS; infection control, PBS; wild-type T7 group, wild-type T7 delivering 1 × 10^7^ PFU (MOI = 100 relative to the bacterial dose); and T7-*phoa* group, T7-*phoa* delivering 1 × 10^7^ PFU (MOI = 100). Fish were then observed for another 48 h. The observation intervals and survival-curve analysis were identical to those used in the *G. mellonella* experiment described above. For PhoA activity measurement, an independent cohort of zebrafish (*n* = 6 per group) was sampled at 1 h post-bacteriophage treatment. Fish were anesthetized with MS-222, and peritoneal lavage fluid (PLF) was collected by injecting 10 µL of ice-cold sterile PBS into the peritoneal cavity, followed by gentle abdominal massage and recovery of the lavage using the same microsyringe. PLF was clarified by centrifugation at 4°C, and the supernatant was analyzed using an alkaline phosphatase activity assay.

### *In vivo* quantification of bacteriophage and bacterial burdens in infection models *G. mellonella* and zebrafish

To examine bacterial proliferation during infection, bacterial load assays were performed at 0, 6, 12, 18, and 24 h post infection using three larvae per time point, and each experiment was performed in triplicate. For hemolymph collection, larvae were washed once in 70% ethanol and twice in PBS to minimize surface contamination. The posterior end was excised, and hemocoel contents (hemolymph, fat body, and internal organs) were expelled into prechilled 2 mL conical tubes. For CFU enumeration, a 10 µL aliquot of hemocoel contents was subjected to serial 10-fold dilutions and plated onto MacConkey agar, followed by incubation at 37°C for 12 h. For bacteriophage quantification, the hemocoel contents were clarified at 4°C (4,000 × *g*, 10 min). A 10 µL aliquot of the supernatant was serially diluted and assayed by the double-agar overlay plaque method to determine PFU. Adult zebrafish were euthanized with an overdose of buffered MS-222 (300 mg/L), rinsed once in 70% ethanol and twice in sterile PBS, and processed individually. Whole fish were transferred to 2 mL sterile bead-beating tubes pre-loaded with ceramic beads and homogenized in ice-cold sterile PBS (typically 1 mL per fish) using two 30 s pulses at 4°C. Homogenates were briefly clarified (500 × *g*, 1 min), serially 10-fold diluted in PBS, and 100 µL of each dilution was plated onto MacConkey agar. Plates were incubated at 37°C for 12 h. For bacteriophage enumeration, an aliquot of the same homogenate was clarified at 4°C (4,000 × *g*, 10 min) to remove host cells and debris. The supernatant was then filtered through a 0.22 µm membrane to eliminate residual bacteria. A 10 µL aliquot of the clarified supernatant was serially diluted in PBS, and PFU were determined by the double agar overlay method using the strain appropriate indicator host.

### Immune gene expression in *G. mellonella* and zebrafish

We assessed *in vivo* immune gene expression using real-time quantitative PCR (RT-qPCR). Samples were collected at 6, 12, and 24 h after infection with *E. coli 65*. *G. mellonella* larvae were chilled on ice for 10 min; the posterior end was excised, and hemocoel contents (hemolymph, fat body, and internal organs) were expelled into precooled 2 mL conical tubes. Samples were weighed, and Trizol reagent was added at a ratio of 1 mL per 100 mg tissue. Total RNA was extracted according to the manufacturer’s instructions, as previously described (24). For zebrafish, adult fish were euthanized with an overdose of buffered MS-222 (300 mg/L), and under aseptic conditions, the kidney, spleen, liver, and intestine were dissected. Total RNA was isolated using the same Trizol procedure as above. Subsequently, 1 µg of total RNA was treated with a genomic DNA removal reagent and reverse transcribed to cDNA following the kit protocol (Accurate Biology, China). The cDNA was diluted 10-fold and used for SYBR Green-based qPCR (Accurate Biology, China). Thermocycling conditions were 95°C for 30 s, followed by 40 cycles of 95°C for 5 s and 60°C for 30 s, performed on a Roche LightCycler 480 II. Relative expression levels were calculated using the ΔΔCt method. All primers are listed in the Data S3. Each sample was run in technical triplicate, and experiments were performed in ≥3 independent biological replicates.

## RESULTS

### Construction and *in vitro* functional validation of T7-*nluc* and T7-*phoa*

**Fig 1:**
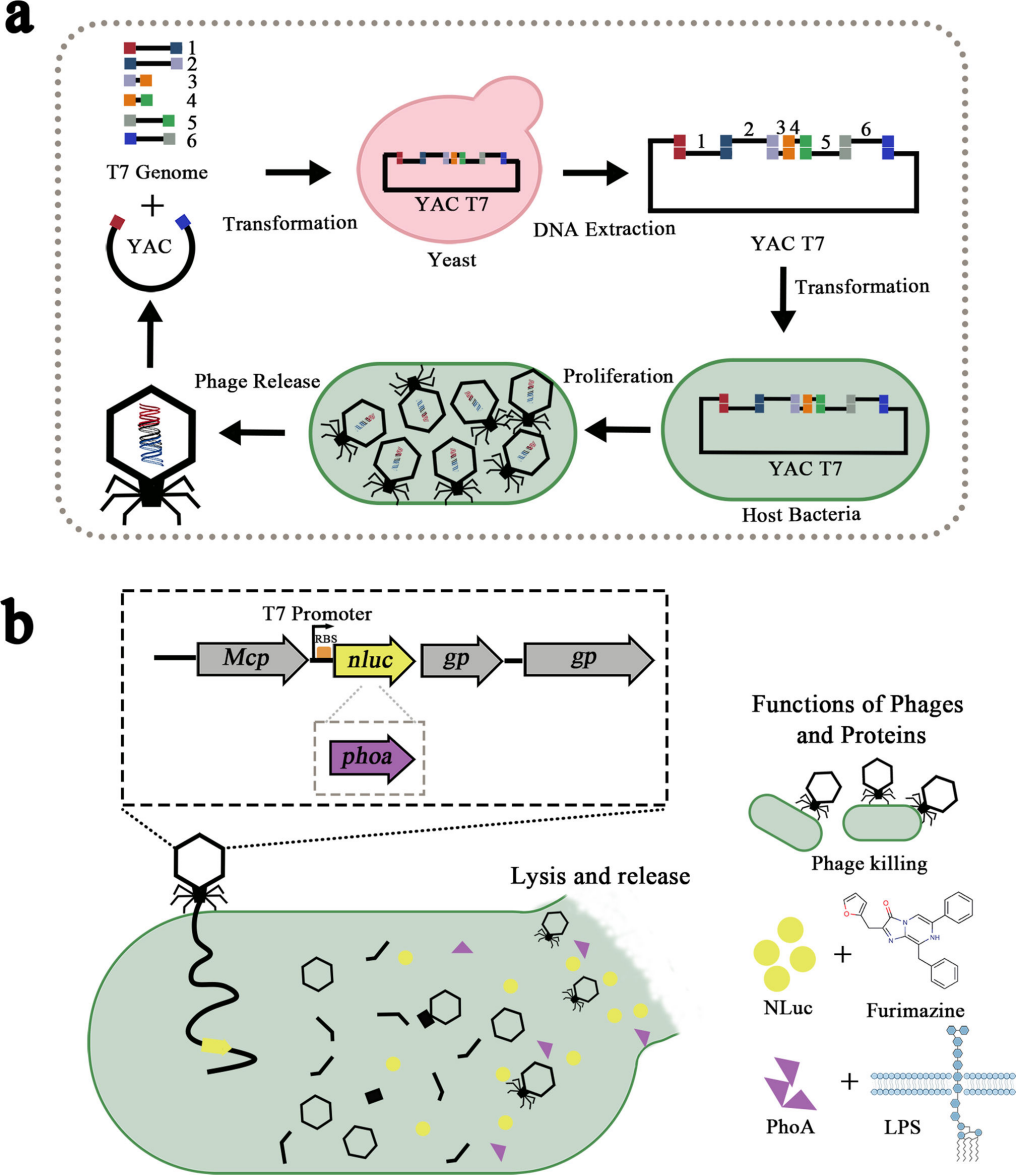
Workflow for constructing engineered bacteriophages. (**a**) PCR products amplified from the bacteriophage genomic DNA were co-transformed into yeast together with a linearized yeast replicon fragment (pRS415) derived from the yeast artificial chromosome (YAC). In yeast, the bacteriophage genome was assembled and captured in the YAC by gap-repair cloning. The resulting YAC-bacteriophage DNA was extracted and transformed into the bacterial host. Infectious bacteriophages were regenerated from the YAC-bacteriophage DNA and formed plaques on lawns of the host bacterium. (**b**) Production and release of the heterologous effector occur after bacteriophage-induced lysis of the host cell. The effector is co-released with progeny virions and reacts with its cognate substrate to exert its functions, including generating bioluminescence and attenuating inflammatory responses. *Mcp*, major capsid protein; *gp*, gene product.

**Fig 2:**
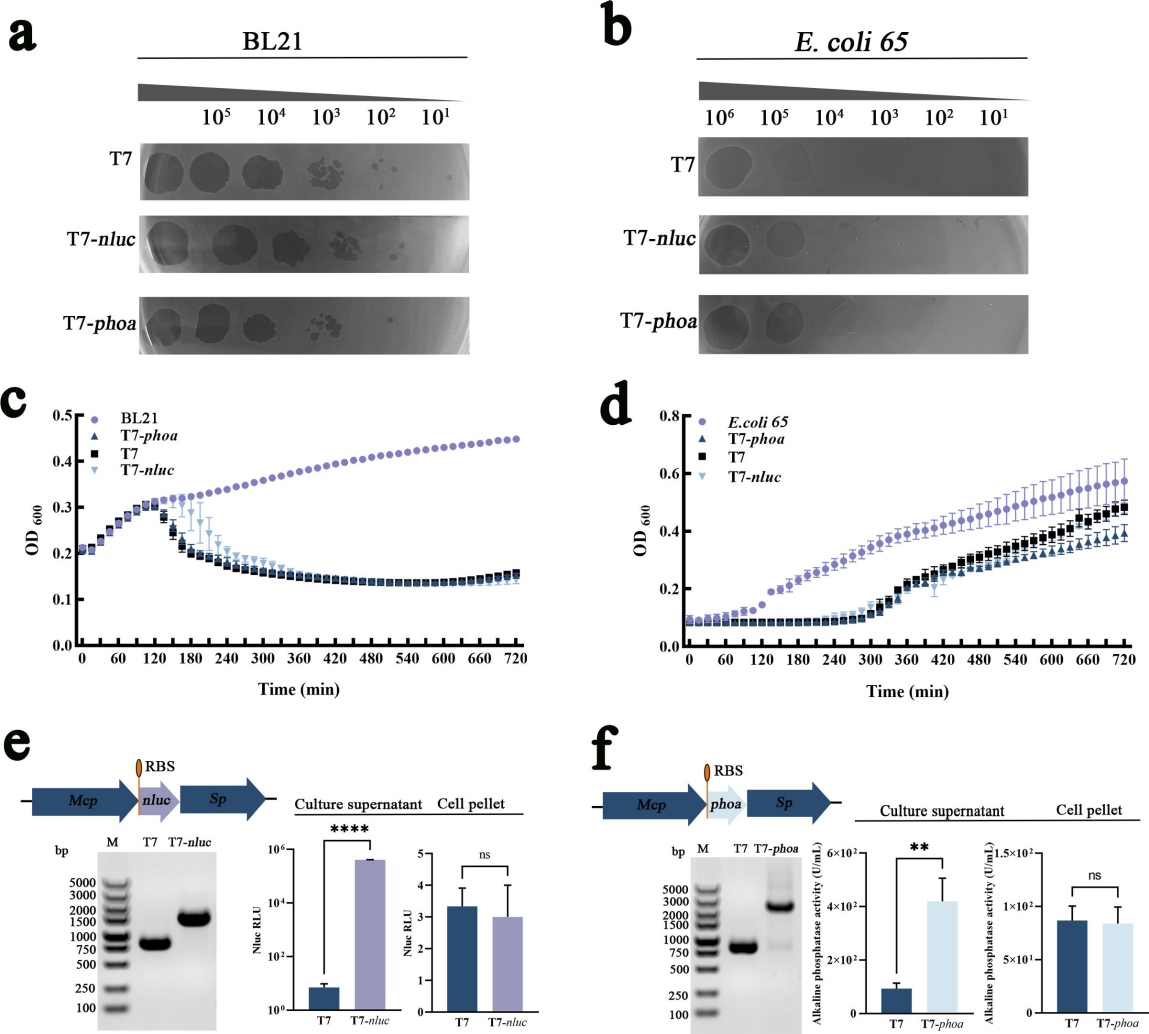
*In vitro* lysis capability and effector activity of the engineered bacteriophage. (**a and b**) Spot-on-the-lawn assay of the engineered bacteriophage at serial dilutions. (**c and d**) Turbidity-reduction assays showing lysis dynamics of wild-type and engineered bacteriophages against BL21 (**c**) and *E. coli 65* (**d**). Log-phase cultures were challenged to a final bacteriophage titer of 1 × 10^4^ PFU/mL, and OD_600_ was recorded every 15 min at 37°C for 12 h. (**e**) The T7-*nluc* construct was verified by PCR using the T7-F and T7-R primers. Supernatant and pellet bioluminescence after infecting *E. coli 65* with T7-*nluc* or T7. After centrifugation, equal volumes of supernatant and resuspended pellet were incubated with Furimazine and luminescence was measured under identical acquisition settings. (**f**) The T7-*phoa* construct was verified by PCR using the T7-F and T7-R primers. Alkaline phosphatase activity in supernatant and pellet fractions after infecting *E. coli 65* with T7-*phoa* or T7, quantified using a commercial alkaline phosphatase assay kit. Data are shown as mean ± SD from three independent experiments. Panels e and f were analyzed by two-tailed unpaired Student’s *t*-test, ****P < 0.0001, **P < 0.01.

We engineered a reporter bacteriophage (T7-*nluc*) and a therapeutic bacteriophage (T7-*phoa*) on a T7 chassis to integrate *in situ* visualization with *in situ* detoxification (Fig. 1a). Initial phenotypic characterization showed no obvious changes in plaque morphology (Fig. 2a and b) or host-cell lysis in BL21 and *E. coli 65* (Fig. 2c and d) relative to wild-type T7, indicating that insertion of the heterologous genes did not impose major fitness costs.

For T7-*nluc*, we selected a strong wild-type T7 promoter and implemented a customized ribosome binding site to maximize reporter output (Fig. 1b). After infection of the clinical isolate *E. coli 65*, supernatants from the T7-*nluc* group showed substantially higher luminescence than those from the wild-type T7 group, whereas pellet-associated signals were similar across conditions (Fig. 2e). These data indicate that T7-*nluc* drives intracellular NLuc expression and releases the reporter into the extracellular milieu during lysis.

T7-*phoa* was designed as a lysis-coupled delivery vehicle in which PhoA is synthesized during infection and released upon host-cell lysis (Fig. 1b). In short-term infections with *E. coli 65*, T7-*phoa* supernatants showed a pronounced increase in alkaline phosphatase activity, whereas T7 supernatants remained near background levels; activity in cell pellets differed only minimally between groups (Fig. 2f). Together, these findings show that T7-*phoa* releases catalytically active PhoA into the infection microenvironment and justify subsequent *in vivo* evaluation.

### Bacterial detection and *in vivo* functional validation of NLuc with T7-*nluc*

To determine whether bacterial persistence could be monitored *in vivo*, we established infection models in zebrafish and *G. mellonella* using the multidrug-resistant clinical isolate *E. coli 65* and administered T7-*nluc* after infection. This reporter bacteriophage was designed to produce and release NLuc during the lytic cycle, enabling sensitive detection by *in vivo* imaging.

**Fig 3:**
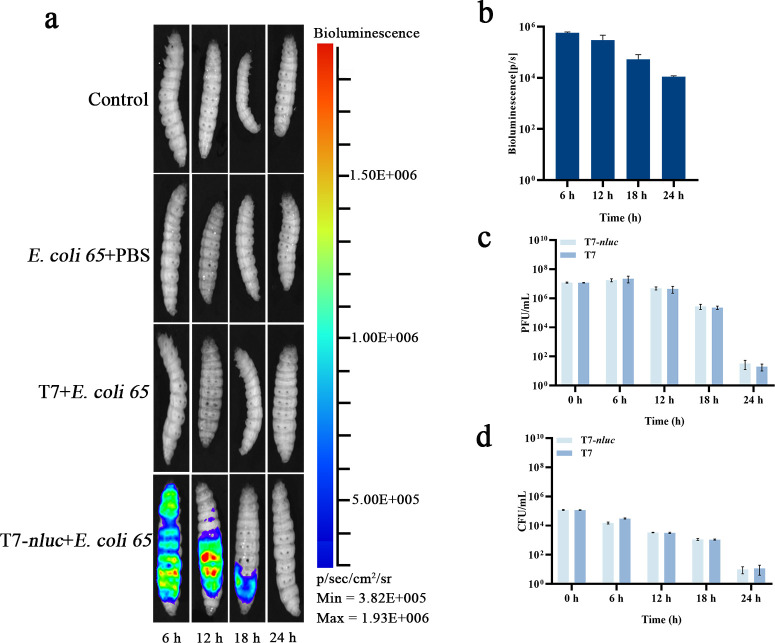
Bioluminescence and bacterial load dynamics after bacteriophage infection in *G. mellonella*. (**a**) Representative bioluminescence images of larvae at 6, 12, 18, and 24 h after bacterial challenge and bacteriophage treatment. Groups: PBS + PBS (blank), *E. coli 65* + PBS (infection control), *E. coli 65* + T7, and *E. coli 65* + T7 *nluc*. Larvae received 10 µL of 1 × 10^7^ CFU/mL *E. coli 65* (or PBS) into the last left proleg, followed 1 h later by 10 µL of 1 × 10^9^ PFU/mL bacteriophage (or PBS) into the last right proleg. At each imaging time point, 2 µL furimazine was injected and photon flux was quantified by ROI analysis using fixed acquisition settings. (**b**) Bioluminescence intensity (p/s) at different time points in T7-*nluc*. (**c**) Bacteriophage titer (PFU/mL) for T7 and T7-*nluc* in hemocoel contents at the indicated time points. (**d**) Bacterial load (CFU/mL) for T7 and T7-*nluc* in hemocoel contents at the indicated time points. Data are presented as mean ± SD (*n* = 3 independent biological replicates). Panel b was analyzed by one-way repeated-measures ANOVA, whereas panels c and d were analyzed by two-way ANOVA, followed by multiple-comparisons testing.

In *G. mellonella*, animals were imaged at 6, 12, 18, and 24 h after bacterial challenge and bacteriophage dosing, with T7, PBS, and infection-only groups as controls. T7-*nluc* produced discrete hotspots at infection-relevant sites, with ROI radiance peaking at 6 to 12 h, decreasing by 18 h, and approaching baseline by 24 h (Fig. 3a and b). Parallel microbiological analysis showed that both CFU and PFU tracked the imaging signal. By 24 h, bacterial counts had dropped to 15 CFU or fewer and luminescence had returned to baseline, indicating progression toward clearance (Fig. 3c and d).

**Fig 4:**
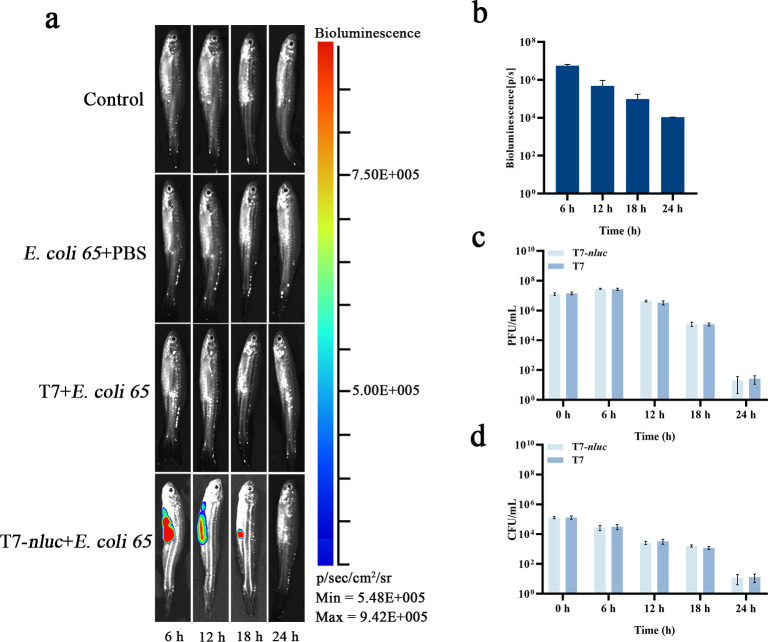
Bioluminescence and bacterial load dynamics after bacteriophage infection in zebrafish. (**a**) Representative bioluminescence images at 6, 12, 18, and 24 h after bacterial challenge and bacteriophage treatment. Groups: PBS + PBS (blank), *E. coli 65* + PBS (infection control), *E. coli 65* + T7, and *E. coli 65* + T7 *nluc*. Adult zebrafish were maintained under standard conditions. Zebrafish were injected intraperitoneally with 10 µL of 1 × 10^7^ CFU/mL *E. coli 65* (or PBS), followed 6 h later by 10 µL of 1 × 10^9^ PFU/mL bacteriophage (or PBS) as assigned. At each time point, 10 µL of furimazine was administered intraperitoneally and bioluminescence was recorded 5 min later under identical instrument settings; photon flux was quantified by ROI analysis. (**b**) Bioluminescence intensity (p/s) at different time points in T7-*nluc* group. (**c**) Bacteriophage titer (PFU/mL) for T7 and T7-*nluc* in homogenized tissues at the indicated time points. (**d**) Bacterial load (CFU/mL) for T7 and T7-*nluc* in homogenized tissues at the indicated time points. Data are presented as mean ± SD (*n* = 3 independent biological replicates). Panel b was analyzed by one-way repeated-measures ANOVA, whereas panels c and d were analyzed by two-way ANOVA, followed by multiple-comparisons testing.

The zebrafish model showed a similar overall pattern. T7-*nluc* again generated well-defined hotspots, ROI radiance was highest at 6 to 12 h, and both tissue CFU and PFU declined markedly by 24 h, when the signal had nearly returned to background (Fig. 4a through d).

Across both hosts, luminescence was temporally concordant with microbiological readouts although the signal peaked earlier and decayed faster in *G. mellonella* than in zebrafish. These results support the use of T7-*nluc* as a practical *in vivo* reporter for tracking viable bacteria and guiding subsequent efficacy analyses.

### Cross model evidence that T7-*phoa* moderates LPS-driven responses while maintaining bacterial clearance

During Gram-negative infection, LPS released from bacterial envelopes can amplify inflammatory signaling. Because excessive inflammation aggravates tissue damage whereas complete suppression may compromise pathogen clearance, we next evaluated whether T7-*phoa* could moderate host responses while maintaining bacterial control.

**Fig 5:**
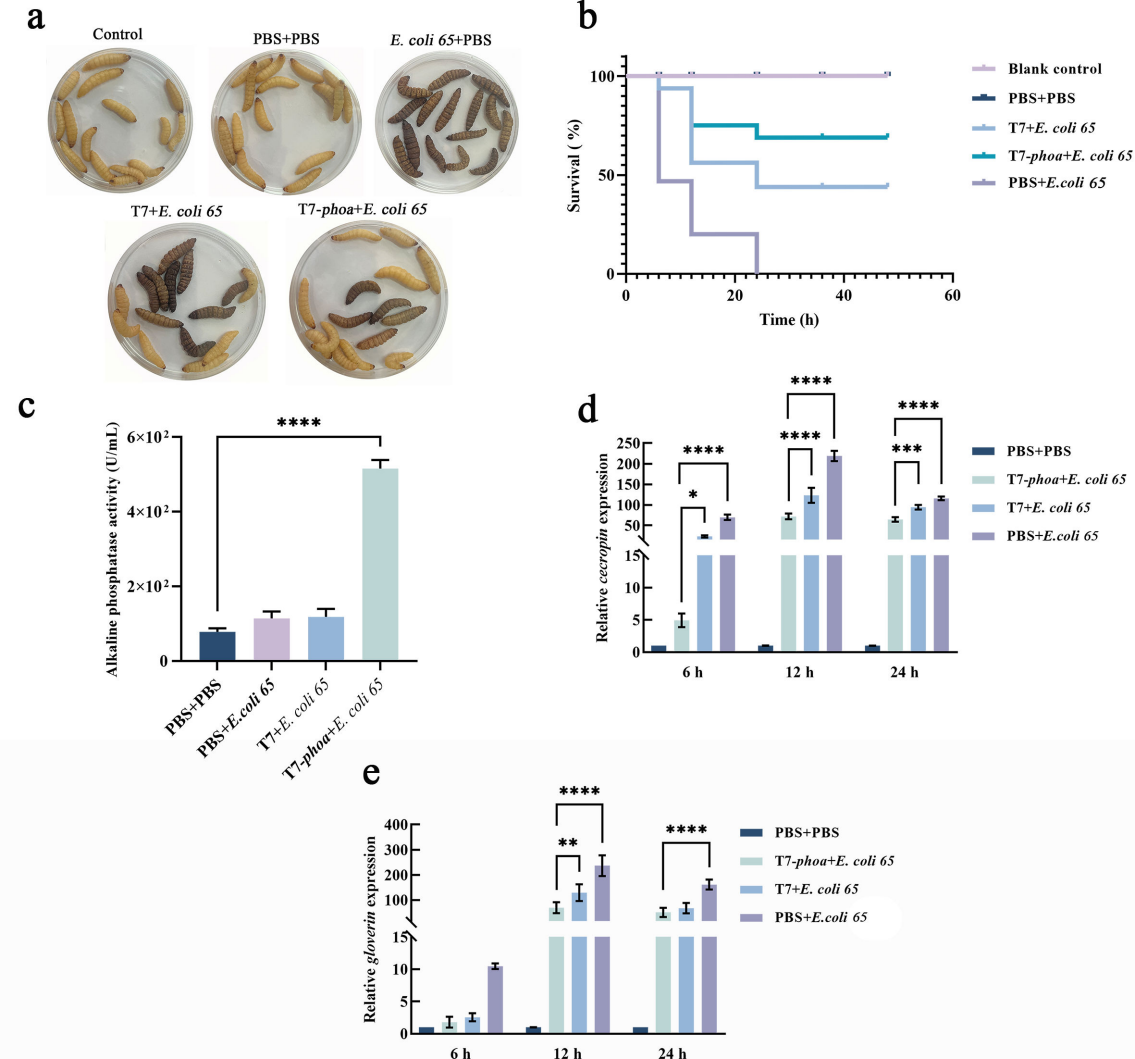
Survival and immune response dynamics in *G. mellonella* post infection. (**a**) Representative larval phenotypes at 48 h after infection under different treatments. (**b**) Kaplan-Meier survival curves of larvae (*n* = 15 per group). Treatment groups are labeled as PBS + PBS, *E. coli 65* + PBS (infection control), *E. coli 65* + T7, and *E. coli 65* + T7 *phoa*. Larvae were challenged with 1 × 10^5^ CFU per larva (10 µL injection) and treated 1 h later with 1 × 10^7^ PFU per larva (10 µL; MOI = 100 relative to the bacterial dose) or corresponding controls. Survival was monitored for 48 h. Statistical analysis: log-rank (Mantel–Cox) test with multiple-comparisons adjustment as indicated. (**c**) Alkaline phosphatase activity in *G. mellonella* hemolymph collected at 1 h after bacteriophage treatment. Groups are as defined for panel **b**. Data are shown as mean ± SD (*n* = 3 independent biological replicates). Statistical analysis: one-way ANOVA with multiple-comparisons correction (Tukey). Significance: ****P < 0.0001. (**d and e**) RT-qPCR analysis of antimicrobial peptide genes *cecropin* (**d**) and *gloverin* (**e**) at 6, 12, and 24 h post infection. Expression levels were calculated using the ΔΔCt method. Data are shown as mean ± SD (*n* = 3 independent biological replicates; each qPCR run in technical triplicate). Statistical analysis: two-way ANOVA with multiple-comparisons correction. Significance: *P < 0.05, **P < 0.01, ***P < 0.001, ****P < 0.0001.

In *G. mellonella*, larvae were challenged with 1 × 10^5^ CFU per larva and treated 1 h later with 1 × 10^7^ PFU per larva (MOI = 100). Kaplan-Meier analysis showed that the T7-*phoa* group consistently had higher cumulative survival than the wild-type T7 group and the infection control group (Fig. 5a and b). Hemolymph collected 1 h after bacteriophage administration also displayed significantly higher alkaline phosphatase activity in the T7-*phoa* group than in the wild-type T7 and PBS groups (Fig. 5c).

We then quantified the antimicrobial peptide genes cecropin and gloverin, which are induced during Gram-negative infection in *G. mellonella*. Both transcripts increased at 6 h relative to the PBS baseline, but their peak levels were lower in the T7-*phoa* group than in the three control groups (Fig. 5d and e), consistent with a dampened infection-induced immune response. Importantly, this attenuation did not compromise bacterial control because no rebound in bacterial load was observed within 24 h and CFU remained comparable to or lower than those in the T7 group at most time points (Fig. 3d).

**Fig 6:**
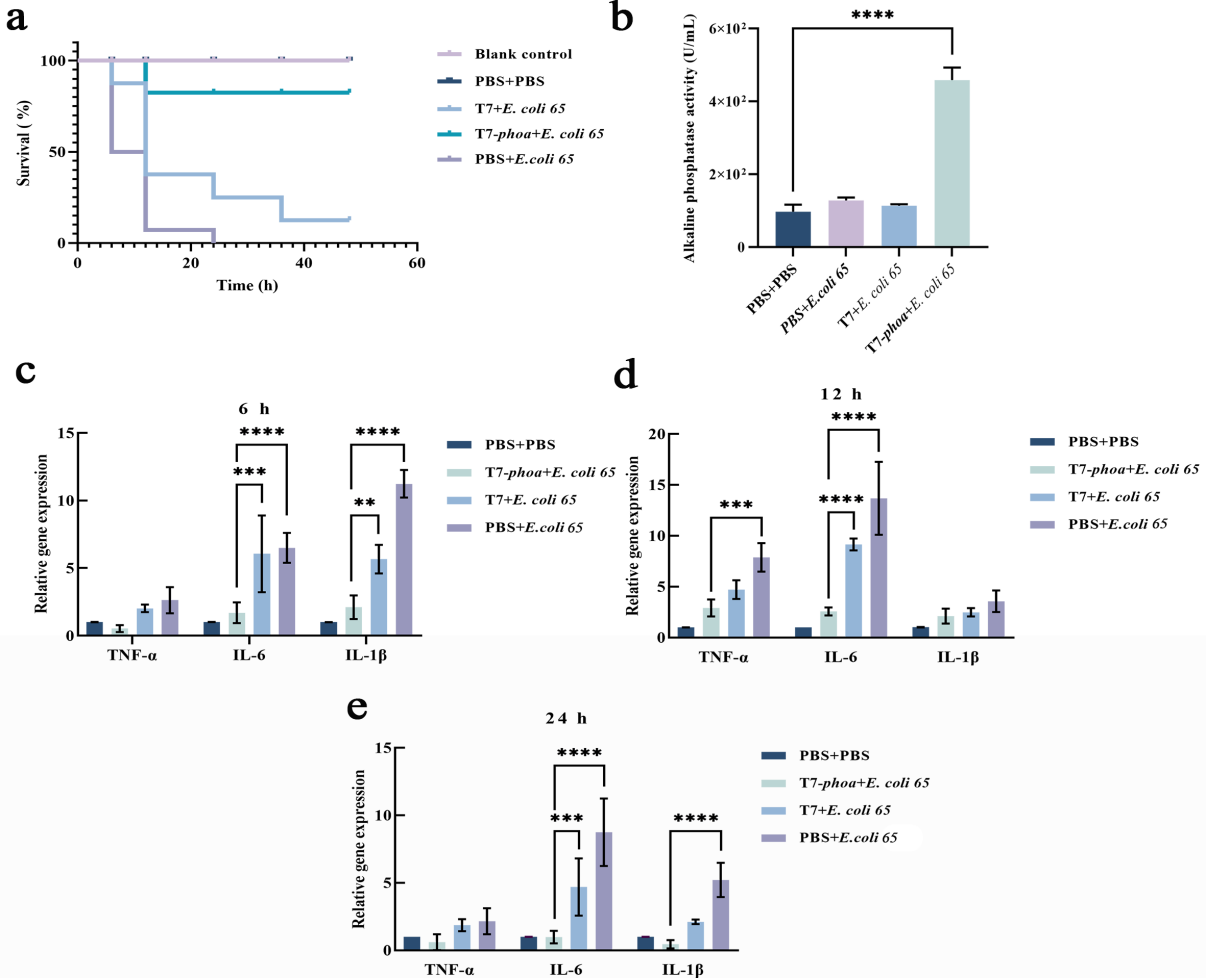
Survival and gene expression analysis in zebrafish after infection. (**a**) Kaplan-Meier survival curves of adult zebrafish (*n* = 15 per group) following *E. coli 65* infection and bacteriophage treatment. Groups are labeled as PBS + PBS, *E. coli 65* + PBS (infection control), *E. coli 65* + T7, and *E. coli 65* + T7 *phoa*. Fish were challenged with 1 × 10^5^ CFU and treated 6 h later with bacteriophages at 1 × 10^7^ PFU (MOI = 100) or corresponding controls, then monitored for 48 h. Statistical analysis: log-rank (Mantel-Cox) test with multiple-comparisons adjustment as indicated. (**b**) Alkaline phosphatase activity in infection-site fluids collected at 1 h after bacteriophage administration. Groups are as defined for panel a. Statistical analysis: one-way ANOVA with multiple-comparisons correction (Tukey). Significance: ****P < 0.0001. (**c–e**) RT-qPCR analysis of inflammatory markers TNF-α, IL-6, and IL-1β in tissues collected at 6, 12, and 24 h post infection. Adult fish were euthanized with buffered MS-222 (300 mg/L) and dissected tissues were collected and homogenized prior to RNA extraction. Data are shown as mean ± SD (*n* = 3 independent biological replicates; each qPCR run in technical triplicate). Statistical analysis: two-way ANOVA with multiple-comparisons correction. Significance: **P < 0.01, ***P < 0.001, ****P < 0.0001. Data are mean ± SD (*n* = 3 independent biological replicates).

Because *G. mellonella* primarily models innate immunity, we next evaluated T7-*phoa* in zebrafish, a vertebrate system with mucosal barriers and adaptive immunity. Using the same bacterial and bacteriophage doses, Kaplan-Meier analysis again showed a clear survival advantage in the T7-*phoa* group over wild-type T7 group and the infection control group (Fig. 6a).

Consistent with lysis-coupled availability *in vivo*, alkaline phosphatase activity was elevated in peritoneal lavage fluid collected 1 h after bacteriophage treatment in the T7-*phoa* group relative to the wild-type T7 and PBS groups (Fig. 6b). RT-qPCR further showed that TNF-α, IL-1β, and IL-6 were broadly induced during the early post-infection window, but their peak amplitudes were significantly lower in the T7-*phoa* group than in the infection control group and T7 groups, while remaining above the PBS baseline (Fig. 6c through e).

From 12 to 24 h, all three inflammatory markers declined, with faster resolution in the T7-*phoa* group than in either control. Tissue CFU also decreased over time, with median values of 20 CFU or fewer at 24 h and no evidence that reduced inflammation was accompanied by increased bacterial burden (Fig. 4d). Together, the two animal models indicate that T7-*phoa* blunts inflammatory peaks and accelerates resolution without measurably impairing bacterial clearance under the conditions tested.

## DISCUSSION

In complex hosts, rapid bacteriophage-mediated killing may come at the cost of stronger inflammatory responses caused by bacteriolysis (25, 26). In this study, T7-*nluc* allowed us to track viable bacteria during infection, while T7-*phoa* was used to test whether lysis-coupled PhoA release could reduce this inflammatory burden. The higher PhoA activity detected after T7-*phoa* treatment in both *G. mellonella* hemolymph and zebrafish peritoneal lavage fluid is in line with this idea. Notably, the T7-*phoa* groups also showed lower and faster-resolving inflammatory responses. Together, these results suggest that engineered bacteriophages can be used not only to reduce bacterial burden but also to make the inflammatory consequences of treatment easier to follow and potentially easier to control.

T7-*nluc* links replication-dependent expression to lysis-dependent release. Because NLuc is produced and released only when infection and amplification occur in the target cell, it provides low-background spatiotemporal mapping of viable bacteria. Reporter bacteriophages carrying NLuc have already shown value for the rapid detection of viable pathogens in clinical matrices (18) and our *in vivo* data extend that logic to animal infection models. In both zebrafish and *G. mellonella*, bioluminescence increased during the early to mid-infection window and declined as bacterial burdens decreased, supporting its use as a companion readout for imaging, redosing, and timing of subsequent mechanistic assays.

Recent progress in bacteriophage and enzyme engineering has focused mainly on improving bactericidal capacity through adsorption, host-range editing (27, 28), lytic performance (29, 30), or combination with adjunct antimicrobials (31, 32). In contrast, the host inflammatory trajectory is less often monitored in parallel.

As a result, peak endotoxin exposure and host response costs are often inferred rather than measured. The detoxification arm targets lipid A at the interface between the site of lysis and the bacterial surface. Localizing PhoA to this immediate milieu is intended to ensure that detoxification occurs spatiotemporally with killing, shortening exposure to peak inflammation and limiting tissue cost. This concept aligns with the intestinal role of PhoA, which dampens TLR4 signaling and supports gut homeostasis (33, 34). Evidence in zebrafish further shows that PhoA lowers intestinal inflammation and LPS burden, aligning with the peak blunting with faster resolution phenotype that we observe in the same species (35). Endogenous detoxification also includes AOAH-mediated deacylation. Loss of AOAH prolongs inflammation and impairs barrier function, underscoring the value of chemically mitigating LPS (36–38).

In our *in vivo* models, the T7-*phoa* group showed lower peaks and faster resolution of inflammatory mediators than wild-type T7 (Fig. 5d and e; Fig. 6c through e), with no evidence of an inflammation-burden rebound trade-off within 24 h relative to bacterial-load metrics (Fig. 3c and d; Fig. 4c and d). Collectively, these findings indicate that T7-*phoa* accelerates inflammatory resolution without compromising bacterial clearance. By coupling bacterial killing with locally available phosphatase activity following on-target lysis, this approach establishes an engineerable link between bacterial control and inflammation control in contrast to the traditional strategy of reducing endotoxin content only prior to administration (33, 39, 40). In the broader set of bacteriophage engineering tools, DspB (41), engineered endolysins (42), and decapping enzymes (43) mainly strengthen the arm that controls bacteria. Without altering lytic kinetics, we add modulation of the host response to lower inflammatory peaks. This yields an integrated strategy to kill, detoxify, and protect the barrier and fills a gap in managing peak inflammation. These efforts are compatible and reinforce one another.

We adopted a modular two bacteriophage regimen in which the reporter bacteriophage and the detoxification bacteriophage are administered separately. The main rationale was to decouple infection monitoring and anti-inflammatory intervention in terms of dosing and timing, allowing each component to be optimized independently while reducing the potential fitness cost associated with carrying additional payloads in a single bacteriophage genome, thereby minimizing adverse effects on replication kinetics and *in vivo* performance. Although integrating both functions into a single dual-function bacteriophage would be more convenient from a translational perspective, its feasibility depends on balancing payload size, expression strength, and bacteriophage fitness, and efficient PhoA localization and folding may also be limiting. Therefore, future work will optimize the construct and directly compare the two bacteriophage regimen with a single dual-function bacteriophage in animal models in terms of luminescence output, bacterial clearance, and inflammatory readouts.

Our study provides proof of concept that an engineered lytic bacteriophage, T7-*phoa*, can enable lysis coupled availability of PhoA in the local infection milieu, thereby blunting inflammatory peaks and accelerating resolution, without evidence of an inflammation clearance trade-off in two animal models. Several considerations remain for future application. First, host and environmental variables may influence efficacy and readouts: although we obtained consistent results under standardized conditions in zebrafish and *G. mellonella*, factors such as diet, medications, microbiota composition, and inter-individual variation in mammals could alter bacteriophage replication, substrate biodistribution, and response thresholds, and thus warrant systematic evaluation. Second, generalizability across strains and Gram-negative pathogens remains to be established. This proof-of-concept study relied on a single clinical isolate (*E. coli 65*), thereby defining the feasibility and therapeutic potential of the approach in this strain background. More broadly, the T7 platform is inherently strain-specific, so therapeutic performance will depend on the availability of susceptible clinical strains within a target population. Accordingly, future work will evaluate both T7-*nluc* and T7-*phoa* across a larger panel of clinical *E. coli* isolates and additional Gram-negative pathogens using matched *in vitro* and *in vivo* endpoints and will broaden coverage through established strategies such as bacteriophage cocktails, prescreening and prioritization of broad host-range bacteriophages, and engineering of receptor-binding proteins or tail fibers to expand host range (44–46). Third, ecological safety and host interactions warrant continued monitoring. Within the scope of our assays and observation windows, we did not detect overt microbiome disruption; however, direct bacteriophage interactions with mucosal surfaces, innate immune sensing, and epithelial barrier integrity should be quantified more explicitly, for example, by assessing mucosal retention, local cytokine signatures, and permeability or tight-junction readouts (47, 48). Fourth, while our *in vivo* phenotype is consistent with PhoA-enabled local LPS detoxification, direct evidence of lipid A dephosphorylation *in vivo* remains to be established using mass spectrometry or dedicated endotoxin bioactivity assays. Accordingly, follow-up studies will prioritize MS-based lipid A profiling and standardized endotoxin activity measurements to characterize the extent and time course of lipid A modification at the infection site. In addition, translation to mammals will require refinement of mechanistic and formulation parameters, and the thresholds for inflammatory responses to lipid A modification and LPS-driven signaling may vary across species, tissues, and infection contexts (49, 50). Therefore, PhoA expression levels and kinetics, tissue distribution, and the corresponding pharmacodynamic and safety windows should be systematically delineated in mammalian models.

For translation, we propose combining pre-administration endotoxin reduction at the formulation level with lysis-coupled, local *in situ* detoxification enabled by T7-*phoa*, paired with conventional antibiotics to build a multilayer strategy that controls bacteria, detoxifies LPS, and protects the barrier in high-burden, high endotoxin infections. In this framework, T7-*nluc* serves as a companion readout to align dosing and imaging timing rather than as a therapeutic. Overall, T7-*phoa* co-localizes bacterial killing and inflammation control in space and time and is supported across experimental layers. With cross-species calibration of thresholds and timing, this approach may offer an engineerable, scalable route to achieve peak blunting with preserved clearance in complex infections.

## Data Availability

The data sets generated during this study are fully available within the article and supplemental material.

## SUPPLEMENTAL MATERIAL

Supplemental tables aem.02303-25-s0001.xlsx Tables S1 to S3.

ASM does not own the copyrights to Supplemental Material that may be linked to, or accessed through, an article. The authors have granted ASM a non-exclusive, world-wide license to publish the Supplemental Material files. Please contact the corresponding author directly for reuse.
